# Bisphosphonate therapy for spinal osteoporosis in Hajdu-Cheney syndrome – new data and literature review

**DOI:** 10.1186/s13023-018-0795-5

**Published:** 2018-04-04

**Authors:** James F. H. Pittaway, Christopher Harrison, Yumie Rhee, Muriel Holder-Espinasse, Alan E. Fryer, Tim Cundy, William M. Drake, Melita D. Irving

**Affiliations:** 1 Department of Endocrinology, St Bartholomew’s Hospital, West Smithfield, London, EC1A 7BE UK; 2 Department of Clinical Genetics, Alder Hey Children’s NHS Foundation Trust, E Prescot Rd, Liverpool, L14 5AB UK; 3 Department of Internal Medicine, Severance Hospital, Endocrine Research Institute, Yonsei University College of Medicine, 50-1 Yonsei-ro, Seodaemun-gu, Seoul, Republic of Korea; 4 Department of Clinical Genetics, Guy’s and St Thomas’ NHS Foundation Trust, Great Maze Pond, London, SE1 9RT UK; 5 Department of Clinical Genetics, Liverpool Women’s NHS Foundation Trust, Crown Street, Liverpool, L8 7SS UK; 6 Department of Medicine, University of Auckland, 85 Park Rd, Grafton, Auckland, 1023 New Zealand

**Keywords:** Bisphosphonates, Diseases, Genetic disorders, Human studies, Dual energy x-ray absorptiometry (DEXA)

## Abstract

**Background:**

Hajdu-Cheney syndrome (HCS) (#OMIM 102500) is a rare, autosomal dominant condition that presents in early childhood. It is caused by mutations in the terminal exon of *NOTCH2*, which encodes the transmembrane NOTCH2 receptor. This pathway is involved in the coupled processes of bone formation and resorption. The skeletal features of HCS include acro-osteolysis of the digits and osteoporosis commonly affecting vertebrae and long bones. Fractures are a prominent feature and are associated with significant morbidity. There is no specific treatment, but with both acro-osteolysis and generalized osteoporosis, it is possible that anti-resorptive treatment might be of benefit. However, to date only a few case reports have evaluated the effectiveness of bisphosphonate treatment.

**Methods:**

We describe the clinical features, treatment regimens and response to bisphosphonate treatment in 7 newly described patients aged 6–39 with HCS, and pooled the data with that from 8 previously published cases (a total of 17 courses of treatment in 15 individuals).

**Results:**

The mean lumbar spine bone mineral density (BMD) z-score before treatment was − 2.9 (SD 1.2). In 14 courses of treatment (82%), there was an increase in BMD with bisphosphonate treatment, but the impact (in terms of change in spinal BMD z-score) appeared to be less with advancing age (*p* = 0.01). There was no evidence that acro-osteolysis was prevented.

**Conclusions:**

Although individual response is variable and age-related, the data support a role for bisphosphonates in preventing or treating spinal osteoporosis in HCS, but bone loss from the lumbar spine may be rapid after cessation.

## Background

Hajdu-Cheney syndrome (HCS) (#OMIM 102500) is a rare, autosomal dominant condition associated with a distinctive skeletal phenotype that includes both generalized osteoporosis and localized acro-osteolysis [1, 2]. It presents in early childhood with characteristic dysmorphic facial features and variable congenital abnormalities, such as polycystic kidneys and heart disease [3–5]. Vertebral and long bone fractures, a consequence of the osteoporosis, are common. Other skeletal findings include premature tooth loss, fibular deformities, scoliosis, joint hyperlaxity, platybasia and basilar invagination [6]. HCS is caused by mutations in the terminal exon of *NOTCH2*, which encodes the transmembrane NOTCH2 receptor [7]. Despite its dominant inheritance many cases do not have a family history as the new mutation rate is high (55%). Signaling through the NOTCH pathway is responsible for cell differentiation and, specifically in the precisely coupled processes of bone formation and bone resorption [8]. In vitro studies suggest the NOTCH pathway plays a role in committing mesenchymal stem cells to the osteoblast lineage, thereby stimulating osteoclastogenesis [9].

Recurrent vertebral and non-vertebral fracture is a major cause of morbidity in patients with HCS and the neurological consequences of progressive platybasia are a significant contributor to the premature mortality associated with the condition. Biochemical and histomorphometric evidence suggests that a high bone turnover state underlies the acro-osteolysis and spinal osteoporosis [10, 11]. A recent histomorphometric study has emphasized marked cortical osteopenia underlying the liability to fracture [12].

A number of clinicians have trialed bisphosphonate therapy in an attempt to retard acro-osteolysis and vertebral bone loss. Only a few case reports of bisphosphonate treatment in HCS have been published [5, 12–14] and with such a rare syndrome, the potential exists for individual anecdote unduly to affect clinical decision-making.

Here, we describe the outcome of bisphosphonate treatment in seven additional patients with HCS, pooled from different centers worldwide. Incorporating data from the previously published case reports, we have attempted to provide useful information on the effectiveness of bisphosphonate treatment on acro-osteolysis and osteoporosis in HCS.

## Methods

Seven patients (5 female; median age 13 years, range 6–39) were studied retrospectively. All carried a diagnosis of HCS based on typical clinical and radiographic features, and five had genetic confirmation. Three of the seven were included in a report that described the presence of *NOTCH2* mutations in the syndrome [7]. The clinical indications leading to the decision to trial bisphosphonate were recurrent fracture and/or declining bone mineral density (BMD), measured by dual-energy X-ray absorptiometry (DXA). A variety of regimens were used but bisphosphonate therapy was given for a minimum of 1½ years. The treatment regimens for individual patients are given in Table 1. One subject (Case 2) was studied for two separate periods. She had treatment with intravenous pamidronate aged 8 to 10, then had sequential BMD scans but no treatment until age 24 when she was treated with intravenous zoledronate. We have also reviewed data from eight other cases that have been described in recent papers [5, 12–15].

**Table 1:** Clinical features, treatment regimens and response to bisphosphonate treatment

Case	Gender- Age (yr)^a^	*NOTCH2* mutation	Fracture Details	Treatment Regimen	Scan interval (years)	Lumbar spine z-score		Acro-osteolysis	
						Before	After	Before	After
1	F 6	c.7198C > T p.R2400X	Metatarsal	Pamidronate 3 mg/kg/3 m	6	− 3.1	− 1.3	Right hand	No response
2^b^	F 8	p.Pro2149Argfs + 2×	Metatarsals, long bones, platybasia	Pamidronate 3 mg/kg/4 m for 1½ yr	1½	−1.7	− 0.3	Hands	Worse
	F 24			Zoledronate 5 mg × 1	2½	−2.9	− 2.5	Hands	Not recorded
3	F 11	Clinical	Vertebral	Alendronate 35 mg/wk. (2 yr) then 70 mg/wk	5	−5.6	−2.6	Not recorded	Not recorded
4	F 15	c.6387delT p.S2129RfsX7	Vertebral, basilar invagination	Pamidronate 3 mg/kg/3 m	3	−4.4	−4.5	Hands and feet	No response
5	F 35	Clinical	None	Zoledronate 5 mg/yr	4	−3.2	−2.8	Not recorded	Not recorded
6	M 36	c.6272delT p.F2091SfsX4	Vertebral compression	Alendronate 10 mg/day	6	−3.0	−4.5	Hands and feet	No response
7	M 39	p.Pro2150fs	Vertebral biconcavity, metatarsal	Pamidronate 30–60 mg/3 m (8 yr); 2 yr. no Rx, then Zoledronate 4–5 mg/6 m (5 yr)	8½	−3.6	− 3.0	Hands and feet	Hands worse - feet no change

^a^At start of treatment, ^b^Patient studied in two separate periods

When not specifically stated, treatment regimens were continued for the entire duration of the scan interval

Progression of acro-osteolysis was assessed from serial radiographs of the hands and feet. BMD at the lumbar spine was measured by dual-energy x-ray absorptiometry. In each case the same machine was used for initial and follow-up scans.

The densitometers used to measure BMD were as follows: GE Lunar Prodigy Advance (Lunar, Madison, WI, USA; cases 1 and 4), Lunar DPX-L (Lunar, Madison, WI, USA; cases 2 (aged 8) and 7), Hologic QDR-4500A (Hologic, Waltham, MA, USA; case 3), GE Lunar Prodigy (Lunar, Madison, WI, USA; cases 2 (aged 24) and 5), Hologic 2000 (Hologic, Waltham, MA, USA; case 6).

Because different densitometers were used and the wide age range we report the BMD results as age-related SD scores (z-scores), compared to the manufacturers’ normal ranges. Mean values were compared by paired t-test and correlations by the Pearson method using IBM SPSS Statistics (version 24.0 for Windows).

## Results

**Fig. 1:**
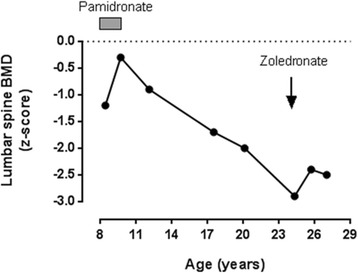
Sequential changes in lumbar spine z-score in one subject (case 2), treated with pamidronate from 8 to 10 years of age, and zoledronate at age 24. The increment in z-score was lower when she was treated as an adult. Between these two treatment periods her BMD z-score fell by 2.6 SD over 14 years

Table 1 summarizes the clinical data, including the age at starting treatment, the regimen given and its duration. Six of the seven subjects had suffered fractures. The mean z-score, measured after a median 4½ years’ bisphosphonate treatment, had increased from − 3.4 (SD 1.2) to − 2.7 (1.4), but this difference was not statistically significant (*p* = 0.158). The response however appeared greater in the young. This was illustrated in subject 2, whose spinal BMD z-score increased by 1.4 SD when treated with pamidronate from 8 to 10 years of age, but when treated with zoledronate at age 24 had only a 0.4 SD increase over a comparable time. In the interval between the two treatment periods, despite enjoying reasonable health and reaching a normal height, her BMD z-score declined markedly with a loss of 2.6 SD over 14 years (Fig. 1).

**Fig. 2:**
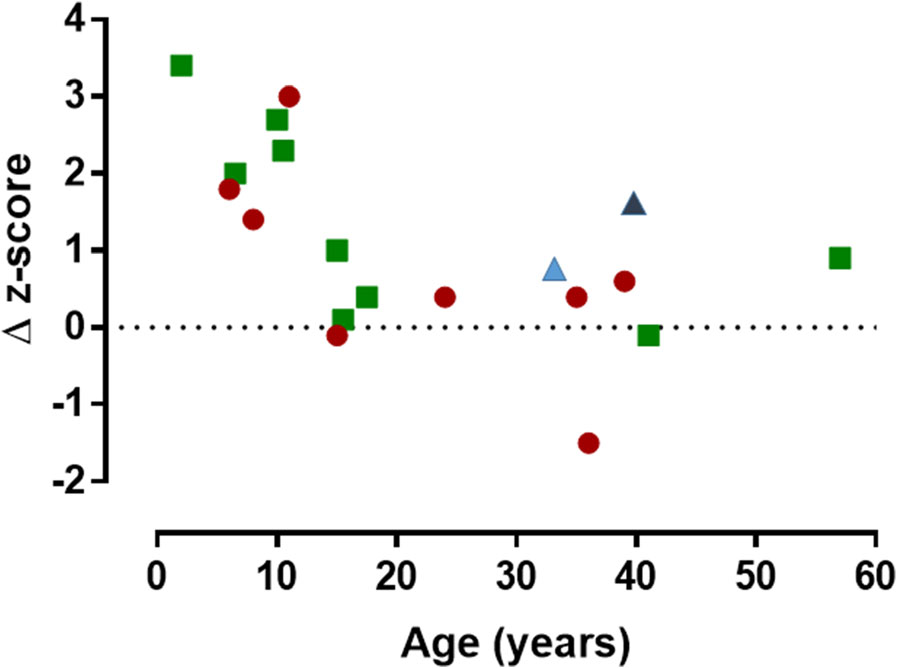
Scatterplot showing the negative correlation between change (Δ) in lumbar spine BMD z-score with bisphosphonate treatment in relation to the age at which treatment was started (17 courses of treatment in 15 individuals). Pearson correlation coefficient − 0.624 (*p* = 0.01). The new cases (from Table 1) are shown by green circles and the cases previously described in the literature (Table 2) are shown by red squares. The light blue triangle indicates the response in the patient described by Adami et al. [17] who was treated with denosumab for 2 years; the dark blue triangle the patient described by McKiernan [18] who was treated with both pamidronate and teriparatide for two years

Table 2 (cases 8–15) lists data extracted from eight other published reports [5, 12–15]. One subject (case 9 [12],) had two separate periods of bisphosphonate treatment evaluated. Including these in the analysis, there was a strong inverse relationship between change in spine BMD z-score and the age at which treatment was started (Pearson correlation coefficient − 0.624, *p* = 0.01; Fig. 2). The only person > 15 years old whose z-score increased by > 0.6 was a 57 year-old woman (case 15 [13],), in whom accelerated bone loss post-menopause may have been a factor.

**Table 2:** Clinical features, treatment regimens and response to bisphosphonate treatment - previously published cases

Case [Ref]	Gender - Age (yr)^a^	*NOTCH2* mutation	Fracture Details	Treatment Regimen	Scan interval (years)	Lumbar spine z-score		Acro-osteolysis	
						Before	After	Before	After
8. [5]	M 2	c.6909dup p.I2304HfsX9	Metatarsals	Pamidronate 3 mg/kg/3 m	5	−4.1	− 0.7	Hands and feet	No response
9.^b^ [12]	M.6½	p.Val2221Glufs*	Long bones	Pamidronate 1 mg/kg/3 m - 1 yr Alendronate 35–70 mg - 5½ yr	6	−1.9	+ 0.1	Hands and feet	No response
	M.17½			Zoledronate 5 mg × 1 age 17½	2½	−2.1	−1.7	Hands and feet	Not recorded
10 [15].	F.10	Clinical	Vertebral, metatarsal, metacarpal	Pamidronate 4 mg/kg/3 m	2	−3.4	−0.7	Hands and feet	Not recorded
11 [12].	M.10½	Clinical	Vertebral, long bones	Alendronate 35–70 mg/wk	4	− 0.5	+ 2.8	Hands and feet	Slowed progression
12 [12].	F.15	p.Leu2301*	Vertebral, long bones	Zoledronate 50 μg/kg/6 m	3	−3.4	−2.4	Hands and feet	No response
13 [12].	M.15½	pGln2263*	Vertebral	Zoledronate 50 μg/kg/6 m	1½	−0.8	− 0.7	Hands and feet	Not recorded
14 [14].	F 41	c.6854delA p.Q2285RfsX9	None	Zoledronate 5 mg/yr	3	−2.5	−2.6	Hands	Worse
15 [13].	F 57	Clinical	Vertebral compression	Alendronate 10 mg/day	4	−3.6	−2.7	Hands	No response

^a^At start of treatment, ^b^Patient studied in two separate periods

*translation termination codon (stop codon)

**Fig. 3:**
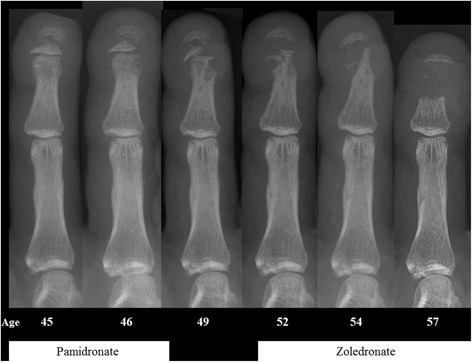
Progression of acro-osteolysis despite treatment with bisphosphonates. Radiographs of the right index finger of subject 7, taken over 12 years. The timing of bisphosphonate treatment is indicated

Changes in acro-osteolysis in the hands were inconsistently reported. In 17 treatment periods (in 15 people) no data was reported in six; in 7 cases there was no response, in three cases progression was documented and in only one case was progression said to have ‘slowed’. There were no documented cases of improvement. Figure 3 illustrates the progressive radiographic change over a twelve-year period in subject 7, who had the most prolonged course of bisphosphonate treatment. Loss of dental bone and teeth was not systematically assessed in any of the cases.

## Discussion

HCS is a rare genetic condition, characterized by generalized osteoporosis and focal bone loss (acro-osteolysis and dental) [1, 2]. In the combined group we describe 75% of the patients over the age of 10 had vertebral fractures. A number of previously published cases have suggested that the generalized osteoporosis, as assessed by lumbar spine BMD, is to some degree responsive to treatment using bisphosphonates [12, 15]. In the expanded data set of 17 courses of treatment in 15 people, there was some improvement in lumbar BMD in 14 (82%), but the responses were variable. Some of this variability might be attributable to the use of regimens of varying intensity and duration, but the age at the start of treatment also seems to be important, with greater responses in the youngest patients.

It is to be expected that the growing skeleton will respond best to bisphosphonates, which work in the growing skeleton both by reducing resorption of the secondary spongiosa, so increasing trabecular bone mass, and by inhibiting endosteal bone resorption during the modelling of long bones, so increasing cortical thickness [16]. It is noticeable from Fig. 2 that the response of BMD to bisphosphonates in HCS becomes muted after the age of 15 – well before the age that peak skeletal mass is reached. Most forms of juvenile osteoporosis arise from the failure of bone acquisition rather than accelerated bone loss, but two of the cases illustrate that bone loss after cessation of bisphosphonates can be very rapid in HCS. As shown in Fig. 2, the teenage years and early twenties of case 2 were marked by a substantial loss of BMD (2.6 SD in 14 years), and case 10 (Table 2), reported by Sakka et al. [12], lost 2.2 SD between the ages of 14 and 19. It is not clear if this bone loss could have been prevented with continued bisphosphonate treatment, and clearly this is an important question for future research. There is insufficient data to comment on fracture rate or whether bisphosphonates might prevent platybasia or the loss of dental bone that underlies the characteristic premature tooth loss in HCS.

We found no evidence that bisphosphonate therapy could promote healing of acro-osteolysis, and indeed progression was documented in some subjects. This suggests either a different mechanism underlies the focal bone loss, that is not amenable to osteoclast inhibition, or that there is inadequate bioavailability of bisphosphonate. The data are insufficient to know whether the rate of progression would have been faster without bisphosphonate treatment.

Adami et al. reported the effects of treatment with another inhibitor of bone resorption, the RANKL-inhibitor denosumab, in a 33 year-old woman with HCS who had previously been treated with bisphosphonates and strontium [17]. The change in lumbar spine BMD (+ 0.5 SD over 2 years) was much in line with the results presented in this paper and acro-osteolysis progressed. McKiernan described the effects of combination therapy with an anabolic agent as well as an anti-resorptive agent in a 39 year-old woman with HCS treated for 2 years with both teriparatide and pamidronate. Her lumbar spine BMD z-score improved from − 7.1 to − 5.5, but over the same period steroid treatment (prescribed for a different indication) was withdrawn. There was no change in acro-osteolysis [14]. For comparison, data from these two cases has been included in Fig. 2.

Given that the molecular basis of HCS is now known [7], and that bisphosphonate therapy in is essentially empirical, opportunities to explore treatment strategies around signaling through NOTCH2 should be considered. In HCS, mutations in the terminal exon of *NOTCH2* lead to the creation of a stop codon upstream of the PEST (proline (P), glutamic acid (E), serine (S), and (T) threonine) domain, responsible for ubiquitination and degradation of NOTCH, leading to a persistence of NOTCH2 signaling [7, 16]. In murine models, this has been shown to increase bone resorption due to a direct effect on osteoclast precursors and due to an increase in RANKL by the osteoblast increasing osteoclastogenesis [17].

NOTCH-ligand interactions result in release of the NOTCH intracellular domain (NICD) which translocates to the nucleus and forms a protein complex with recombination signal-binding protein for immunoglobulin κJ region (Rbpjκ) and Mastermind-like to induce transcription of downstream effectors of this signaling pathway (transcription factors of the hairy enhancer of split (HES) gene family, HES and HEY) [18]. Interruption of this pathway promises a more targeted approach to treatment. Possible drug targets in the pathway include inhibitors of the enzyme complexes that cleave the NOTCH extra- and intracellular domains (alpha and gamma secretase, respectively) or antibodies to the NOTCH2 receptor itself. An antibody to NOTCH2 has been shown to reverse the osteopenic phenotype in HCS mutant mice [19]. NOTCH signaling dysfunction is also implicated in the pathogenesis of a number of hematological and solid tumor malignancies. Gamma secretase inhibitors are in clinical trials in such malignancies, and although not specific to NOTCH2, these results could potentially inform novel therapeutic approaches in HCS [20].

## Conclusions

We have described the responses of acro-osteolysis and spinal BMD to treatment with bisphosphonates in a new cohort of patients with Hajdu-Cheney syndrome, and reviewed the previously published literature. Our data suggest that bisphosphonate treatment does not arrest acro-osteolysis, but spinal BMD is improved. The greatest effect is seen in younger patients and bone loss can be rapid when bisphosphonates are discontinued. Although this is the largest group described to date, the numbers are still small, as is typical with such rare diseases, and larger numbers are required to confirm the efficacy of bisphosphonate treatment. Better therapeutic options may be available in the future with increased understanding of the molecular pathogenesis of the condition and the growing experience of gamma secretase inhibitors in clinical oncology.
