# Correction to: Bisphosphonate therapy for spinal osteoporosis in Hajdu-Cheney syndrome – new data and literature review

**DOI:** 10.1186/s13023-019-1084-7

**Published:** 2019-05-10

**Authors:** James F. H. Pittaway, Christopher Harrison, Yumie Rhee, Muriel Holder-Espinasse, Alan E. Fryer, Tim Cundy, William M. Drake, Melita D. Irving

**Affiliations:** 1 Department of Endocrinology, St Bartholomew’s Hospital, West Smithfield, London, EC1A 7BE UK; 2 Department of Clinical Genetics, Alder Hey Children’s, NHS Foundation Trust, E Prescot Rd, Liverpool, L14 5AB UK; 3 Department of Internal Medicine, Severance Hospital, Endocrine Research Institute, Yonsei University College of Medicine, 50-1 Yonsei-ro, Seodaemun-gu, Seoul, Republic of Korea; 4 Department of Clinical Genetics, Guy’s and St Thomas’ NHS Foundation Trust, Great Maze Pond, London, SE1 9RT UK; 5 Department of Clinical Genetics, Liverpool Women’s NHS Foundation Trust, Crown Street, Liverpool, L8 7SS UK; 6 Department of Medicine, University of Auckland, 85 Park Rd, Grafton, Auckland, 1023 New Zealand

**Correction to: Orphanet J Rare Dis**

**https://doi.org/10.1186/s13023-018-0795-5**

After publication of this article [1], it is noticed reference no. 17 was incorrectly provided, details are shown below:

**Original reference no. 17:**

Canalis E, Schilling L, Yee SP, Lee SK, Zanotti S. Hajdu Cheney mouse mutants exhibit osteopenia, increased Osteoclastogenesis, and bone resorption. J Biol Chem. 2016;291(4):1538–51. 10.1074/jbc. M115.685453.

**Correct reference no. 17**:

G. Adami, M. Rossini, D. Gatti, G. Orsolini, L. Idolazzi, O. Viapiana, A. Scarpa, *E. Canalis* Hajdu Cheney Syndrome; report of a novel NOTCH2 mutation and treatment with denosumab Bone, 92 (2016), pp. 150–156.

The author apologizes for the inconvenience caused.
